# New Appliances and Things Medical

**Published:** 1898-01-08

**Authors:** 


### NEW APPLIANCES AND THINGS MEDICAL.

**[We shall be glad to receive, at our Office, 28 & 29, Southampton Street, Strand, London, W.C., from the manufacturers, specimens of all new preparations and appliances which may be brought out from time to time.]**

### TEA AND COCOA.

(Tower Tea Co., 5, Jewry Street, E.C.)

We have received samples of the above from the Tower Tea Company. The celebrated Tower Tea is now so universally known and appreciated that we need say little about it. We find, however, that the sample forwarded to us maintains the same standard of uniformity and excellence which has always characterised previous teas sent us by the same company. The tea is sent out in elegant tin boxes of one pound, and the price is 2s. The cocoa, which is now also being sold by this firm, and which has been sent us by them for examination consists, firstly, of a pure soluble cocoa essence, and secondly, of a prepared cocoa, containing sugar and arrowroot happily combined, so as to form, when mixed with water or milk, a most pleasant and nutritious beverage. Both these samples of cocoa are, *sui generis*, of excellent quality.

### DR. TRESTRAIL'S CARBOLIC DISINFECTANT NIGHT LIGHT.

(Palmer and Co., Limited, Stratford, London, E.)

These are very good night lights. They burn steadily and well, and the flame is not too large. They are of the safe, old-fashioned type which has to be placed in a saucer of water, but the wick is provided with a metal foot-piece, so that it remains upright to the end. Each light is surrounded by absorbent paper containing carbolic disinfectant, which gradually becomes vapourised. This is a matter to which we do not attribute much importance. They are good night-lights; that is the great point.

